# Starburst amacrine cells, involved in visual motion perception, lose their synaptic input from dopaminergic amacrine cells and degenerate in Parkinson’s disease patients

**DOI:** 10.1186/s40035-023-00348-y

**Published:** 2023-04-03

**Authors:** Xavier Sánchez-Sáez, Isabel Ortuño-Lizarán, Carla Sánchez-Castillo, Pedro Lax, Nicolás Cuenca

**Affiliations:** 1 Department of Physiology, Genetics and Microbiology, University of Alicante, San Vicente del Raspeig, Spain; 2 Alicante Institute for Health and Biomedical Research (ISABIAL), Alicante, Spain; 3 Ramón Margalef Institute, University of Alicante, San Vicente del Raspeig, Spain

**Keywords:** Parkinson’s disease, Retinal neurodegeneration, Human retina, ChAT amacrine cells, Dopaminergic amacrine cells

## Abstract

**Background:**

The main clinical symptoms characteristic of Parkinson’s disease (PD) are bradykinesia, tremor, and other motor deficits. However, non-motor symptoms, such as visual disturbances, can be identified at early stages of the disease. One of these symptoms is the impairment of visual motion perception. Hence, we sought to determine if the starburst amacrine cells, which are the main cellular type involved in motion direction selectivity, are degenerated in PD and if the dopaminergic system is related to this degeneration.

**Methods:**

Human eyes from control (*n* = 10) and PD (*n* = 9) donors were available for this study. Using immunohistochemistry and confocal microscopy, we quantified starburst amacrine cell density (choline acetyltransferase [ChAT]-positive cells) and the relationship between these cells and dopaminergic amacrine cells (tyrosine hydroxylase-positive cells and vesicular monoamine transporter-2-positive presynapses) in cross-sections and wholemount retinas.

**Results:**

First, we found two different ChAT amacrine populations in the human retina that presented different ChAT immunoreactivity intensity and different expression of calcium-binding proteins. Both populations are affected in PD and their density is reduced compared to controls. Also, we report, for the first time, synaptic contacts between dopaminergic amacrine cells and ChAT-positive cells in the human retina. We found that, in PD retinas, there is a reduction of the dopaminergic synaptic contacts into ChAT cells.

**Conclusions:**

Taken together, this work indicates degeneration of starburst amacrine cells in PD related to dopaminergic degeneration and that dopaminergic amacrine cells could modulate the function of starburst amacrine cells. Since motion perception circuitries are affected in PD, their assessment using visual tests could provide new insights into the diagnosis of PD.

**Supplementary Information:**

The online version contains supplementary material available at 10.1186/s40035-023-00348-y.

## Background

Although, traditionally, Parkinson's disease (PD) has been mainly considered as a motor disorder, it is now thought to affect multiple systems and tissues, with a wide variety of non-motor features [1]. Among them, besides cognitive decline and dementia, mood disturbances [2], sleep disorders [3], and visual symptoms are commonly described in patients [1]. Importantly, visual alterations have a strong negative effect in the daily lives of patients and caregivers, as they are involved in falls, reading or driving difficulties, and other daily routines. Some of the visual symptoms include hallucinations, impairment of visual acuity [4], contrast sensitivity [5, 6], color vision [7, 8] or motion perception [9, 10], and altered pupil reflex [11], electroretinographic response and visual evoked potentials [12, 13]. Also, structural changes and retinal thinning have been observed with optical coherence tomography (OCT) [14]. In addition, direct retinal affection in PD has been already been described, and some of the sleep and visual alterations have been proposed as early biomarkers related to the degeneration of retinal dopaminergic amacrine (DA) cells [15] and intrinsically photosensitive retinal ganglion cells [16], or to the accumulation of phosphorylated alpha-synuclein, similar to Lewy bodies, in the retina [17, 18].

The human visual system constantly perceives and analyzes motion information coming either from moving objects or from self-motion. Detecting and distinguishing motion signals is a fundamental task for the perception of the surrounding environment and following proper response or reflex. The processing of motion perception begins in the retina, where different specialized neurons participate in complex neural circuitries for direction selectivity (reviewed in [19]). Among these neurons, the starburst amacrine cells have been widely demonstrated to play a key role in these circuitries [20, 21].

Starburst amacrine cells are interneurons that express gamma-aminobutyric acid (GABA) and choline acetyltransferase (ChAT) and are present across vertebrates [22–27]. They display a characteristic “mirror” distribution in which their cell body is located either in the inner nuclear layer (INL; OFF ChAT) or in the ganglion cell layer (GCL; ON ChAT), with their dendrites stratified in separate sublayers (stratum 2, sublamina OFF; and stratum 4, sublamina ON, respectively) of the inner plexiform layer (IPL) [26, 28]. Their first description can be tracked back to 1892 when Santiago Ramón y Cajal described small monostratified amacrine cells at the INL with their plexus in S2 stratum, and small monostratified displaced amacrine cells with their plexus in S4 stratum [29].

Because starburst amacrine cells play a crucial role in generating direction selectivity responses to a moving stimulus, and PD patients have motion perception disturbances, we investigated if these cells are affected in the postmortem PD retinas. Also, the degeneration of retinal DA cells has already been reported in PD, and DA cells are known to have a widespread effect on retinal function and regulation [15]. Hence, we wondered whether ChAT amacrine cells are also regulated by DA cells in the human retina.

## Methods

### Source of human tissue and clinical characterization

Post-mortem ocular tissues from human donors were obtained from volunteers, who signed written informed consent, in the Arizona Study of Aging and Neurodegenerative Disorders (AZSAND)/Banner Sun Health Research Institute Brain and Body Donation Program (BBDP; https://www.brainandbodydonationprogram.org [30]) and from the Hospital General Universitario de Alicante (HGUA). All the procedures involving the use of human tissue followed the Declaration of Helsinki (Code of Ethics of the World Medical Association) and were approved by the Ethics Committee of the University of Alicante (UA-2018-04-17). The age of donors ranged from 57 to 93 years, and no ophthalmological pathologies were reported. Two experimental groups were used in this study: PD group, composed of individuals diagnosed with PD (*n* = 9, one eye per subject; 76 ± 2 years old; AZSAND), and control group, composed of healthy individuals (*n* = 10, one eye per subject; 71 ± 2 years old; HGUA). The left eye was used in all cases. The age was not significantly different between groups (Unpaired *t*-test, *P* = 0.0844). The information of each subject related to age, group, and disease stage is summarized in Additional file 1: Table S1.

### Tissue processing

After enucleation, ocular globes were fixed in paraformaldehyde (3.75%–4%) for 2 h at room temperature (RT) or 24–72 h at 4 °C. They were washed and cryoprotected in increasing sucrose solutions (10%–20%–30%). Eyes were further dissected by removing the cornea, iris, lens and vitreous body, and the remaining tissue was cut into eight pieces. From them, some were prepared as wholemount retinas (inferonasal quadrant), and some were cut in a cryostat to obtain cross-sections of 14 microns (temporal-central quadrant).

### Retinal immunohistochemistry

For immunohistochemical staining, retinal tissue was washed three times in 0.1 M phosphate buffer and incubated with diluted primary antibodies for 3 nights, in wholemounts, or overnight, in sections. The used primary antibodies were goat polyclonal anti-ChAT (1:200; AB144P Merck Millipore, Darmstadt, Germany), rabbit polyclonal anti-tyrosine hydroxylase (TH) (1:200; ab112 Abcam, Cambridge, UK), mouse polyclonal anti-parvalbumin (PV) (1:200; P3088 Sigma-Aldrich, Darmstadt, Germany), sheep anti-vesicular monoamine transporter 2 (VMAT2) (1:200; ABS2250 Phospho Solutions, Aurora, CO), rat polyclonal anti-glycine (1:100; gift from Dr. David V. Pow, University of Queensland, Australia), rabbit polyclonal anti-GABA (1:100; gift from Dr. David V. Pow, University of Queensland, Australia), and mouse monoclonal anti-calbindin (1:1000; 300 Swant, Marly, Switzerland). Then, they were washed and incubated with the corresponding secondary antibodies at a dilution of 1:100 during two nights at 4 °C (for wholemounts) or for 1 h at RT (sections). The polyclonal secondary antibodies used were donkey anti-goat conjugated to Alexa-488 (A11055), donkey anti-goat conjugated to Alexa-555 (A32816), donkey anti-rabbit conjugated to Alexa-555 (A210206), donkey anti-rabbit conjugated to Alexa-555 (A31572), donkey anti-mouse conjugated to Alexa-555 (A31570), donkey anti-sheep conjugated to Alexa-633 (A21082), donkey anti-sheep conjugated to Alexa-488 (A11015), and donkey anti-rat conjugated to Alexa 488 (A21208), all from ThermoFisher Scientific (Rockford, IL). Samples were washed again in phosphate buffer and then mounted in Citifluor (Citifluor Ltd, London, UK), coverslipped, and imaged in a Leica confocal microscope TCS SP8 (Leica Microsystems, Wetzlar, Germany).

### Cellular quantification

The number of cholinergic amacrine cells was counted in the inferonasal quadrant of retinal flat mounts. The whole quadrant was imaged, and 8 areas of 1 mm^2^ were randomly drawn every other millimeter through the retinal center to periphery axis. All cells within each counting area were counted and the number of cholinergic amacrine cells per mm^2^ was calculated for the different eccentricities. The number of dopaminergic synaptic contacts into cholinergic amacrine cells was counted in cross-sections of the temporal-central retina. The quantification was performed in 1 mm^2^ areas at 1 mm from the optic nerve to the nasal region and at 1, 3, 5, 7, and 9 mm from the optic nerve to the temporal region. Quantifications were done by two independent researchers and a third one was consulted in case of doubts.

### Single-cell data analysis

Adult human retina single-nucleus RNA-sequencing (snRNA-seq) data were obtained from publicly available datasets deposited in GEO (GSE183684, [31]). We used the three available datasets of control adult humans: aged 25, 50 and 52 years. Sequence read archive (SRA) data were downloaded, transformed to FASTQ files using the SRA toolkit and then processed with the 10× genomics cloud analysis. The output files were processed in R using Seurat (v4.0; [32]) to set up each individual Seurat object with the “read10X” function. Then, the different datasets were merged into one object and further processed. First, data were filtered according to quality control parameters and then normalized, and variable features were identified. Then, data were scaled, and linear dimensional reduction was performed, principal components were determined, nearest neighbors were obtained, and uniform manifold approximation and projection (UMAP) clustering was performed. Expression of known markers of ChAT amacrine cells (ChAT, PAX6 or ELAVL3) was used to identify the ChAT amacrine cell population within all retinal cells in the dataset. Further filtering allowed us to select only the ChAT amacrine cells by using the “subset” function with ChAT expression > 0.5. Again, nearest neighbors were found, UMAP was run, and clusters were identified. DimPlots and VlnPlots were used to represent expression of different genes of interest.

### Data for correlation analyses

For the correlation analyses we used the data of the density of DA cells used in Ortuño-Lizarán et al. [15] and the data of the retinal Lewy-type synucleinopathy (LTS) score in Ortuño-Lizarán et al. [17]. We performed correlation analysis between these parameters and (1) the density of the different ChAT^+^ populations at 2, 4 and 6 mm from the optic nerve and (2) the density of DA-ChAT connections at 1 mm from the optic nerve. We selected those regions for being the most affected ones.

### Statistical analysis

Statistical analysis of the cellular and connectivity quantifications was performed using the GraphPad Prism software (version 8; Graph-Pad Software Inc., San Diego, CA). Statistical significance was determined with a two-way ANOVA with Sidak’s multiple-comparison test. Correlations were performed by a two-tailed Spearman test using the SPSS Statistics software (version 20.0; IBM, Armonk, NY). Results are presented in bar plots showing the mean ± standard error of the mean (SEM), where each dot represents one independent sample. Plots were made using GraphPad Prism software and, in all cases, *P* < 0.05 was considered to be statistically significant.

## Results

### Two distinct ChAT amacrine cell populations can be found in the human retina

**Fig. 1:**
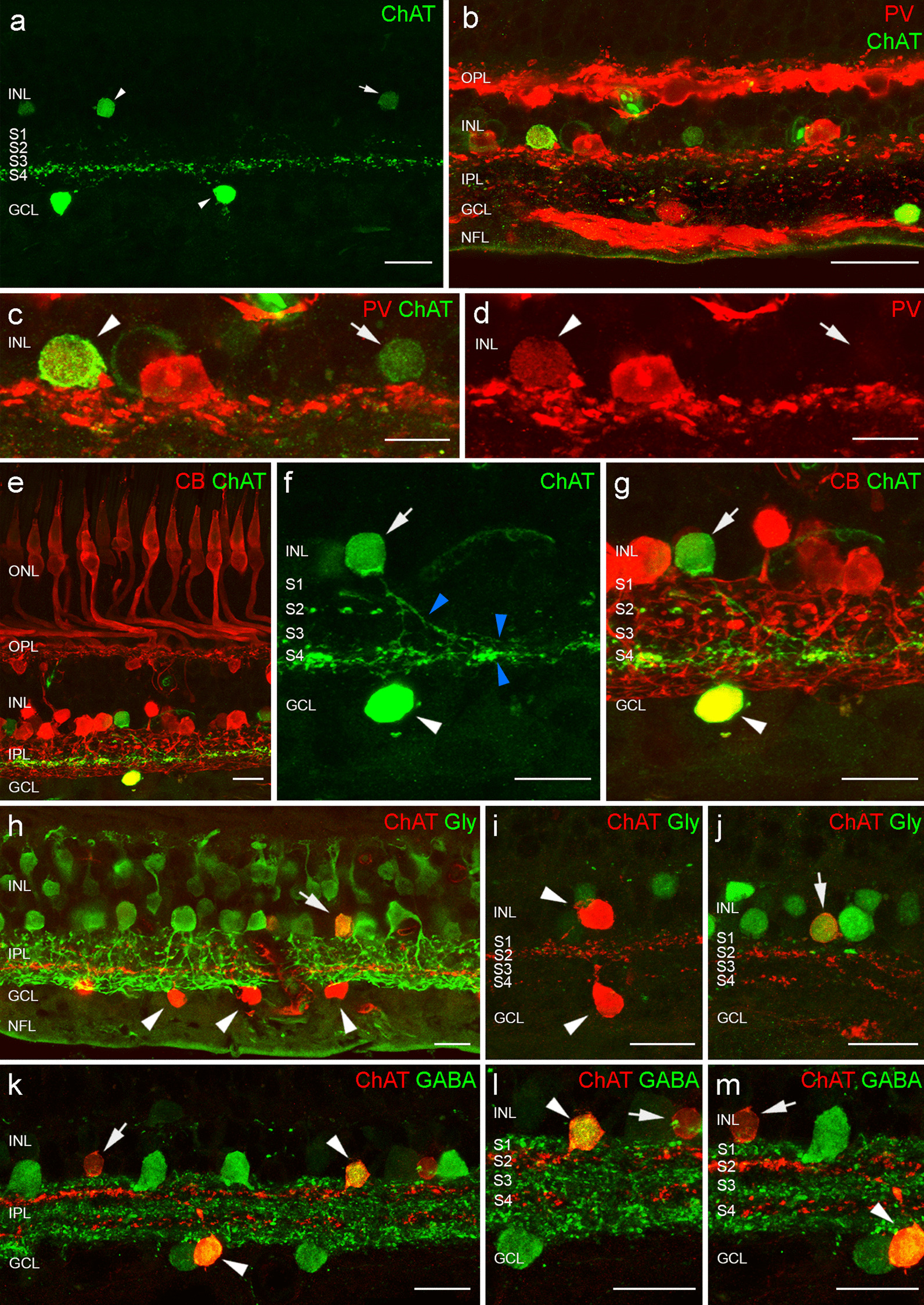
**a** Immunostaining against choline acetyltransferase (ChAT) in cross-sections of human retina. Two types of ChAT-positive cells can be observed: bright (arrowheads) and dim (arrows). **b** Immunostaining against parvalbumin (PV, in red) and choline acetyl transferase (ChAT, in green) in the human retina. **c**, **d** Details of the ChAT-positive cells in the inner nuclear layer (INL). Bright ChAT-positive cells (arrowhead) are also parvalbumin-positive, and dim ChAT-positive cells (arrow) are parvalbumin-negative. **e** Immunostaining against ChAT (green) and calbindin (red) in the human retina. **f**, **g** Details of the ChAT-positive cells in the INL and the ganglion cell layer (GCL). **f** Thin processes (blue arrowheads) of dim ChAT-positive cells (arrow) stratify in strata 3 and 4 (S3/S4, blue arrowheads). **g** Bright ChAT-positive cells (arrowhead) are also calbindin-positive, and dim ChAT-positive cells (arrow) are calbindin-negative. **h–j** Immunostainings against ChAT (red) and glycine (green) in the human retina. Bright ChAT-positive cells (arrowheads) are glycine-negative, and dim ChAT-positive cells (arrow) are also glycine-positive. **k**–**m** Immunostainings against ChAT (red) and GABA (green) in the human retina. Bright ChAT-positive cells (arrowheads) are also GABA-positive, and dim ChAT-positive cells (arrows) are GABA-negative. S2: stratum 2 in the inner plexiform layer (IPL), S4: stratum 4 in the IPL, OPL: outer plexiform layer, NFL: nerve fiber layer. Scale bars: 20 μm (**a**–**d**; **i**–**m**), 15 μm (**e**–**h**)

In humans, cholinergic amacrine cells in the retina have their cell bodies located in the INL and the GCL and they mainly stratify in the S2 and S4 of the IPL, respectively (Fig. 1a) [25, 26]. Although ChAT-containing amacrine cells are usually considered the starburst amacrine cells, another type of ChAT amacrine cell has been described in other species [22, 24, 27, 33]. In them, the two types of ChAT cells have been distinguished by their different expression of calcium-binding proteins. Here, we show two different patterns of ChAT immunoreactivity intensity in the human retina (Fig. 1a): bright (arrowheads) and dim (arrows). We wondered if this pattern of staining was unveiling two different ChAT amacrine populations or if it was showing different ChAT levels on the same type of cell. To explore this possibility, we performed double immunostaining for ChAT (green) and parvalbumin (red) in retinal sections (Fig. 1b–d) and observed that only the bright ChAT amacrine cells, but not the dim ones, expressed parvalbumin. We also performed double immunostaining for ChAT (green) and calbindin (red) in these sections (Fig. 1e–g) and the same pattern was repeated: only the bright ChAT amacrine cells, but not the dim ones, expressed calbindin. These results support the evidence that they are two different types of amacrine cells. Interestingly, all the dim ChAT cells were located at the INL (Fig. 1c, d), and they were usually placed some rows more external compared to the bright cells. Due to their low staining intensity, it was difficult to find dendrites emerging from their somas, although in some cases we found thin processes that stratify in S3 and S4 strata (Fig. 1f, blue arrowheads). In contrast, the bright cells were located both at the INL and GCL (Fig. 1a–g). Moreover, knowing that amacrine cells may be classified into glycine-positive populations and GABA-positive populations, we carried out double immunostaining for ChAT (red) and glycine (green) (Fig. 1h–j) and for ChAT (red) and GABA (green) (Fig. 1k–m) in retinal sections. We observed that dim (Fig. 1h–j, arrows), but not bright (Fig. 1h–j arrowheads), expressed glycine; and that bright (Fig. 1k–m arrowheads), but not dim (Fig. 1k–m arrows), expressed GABA. Preliminary results using deposited available snRNA-seq data from adult human retina (Additional file 1: Fig. S1) show two clusters of ChAT amacrine cells that would correspond to these two populations: bright and dim (Additional file 1: Fig. S1e). Gene expression at the single-cell level was consistent with what we observed with immunostaining: all cells expressed ChAT (Additional file 1: Fig. S1f, g), bright ChAT cells expressed calbindin, parvalbumin, and GABA transporter 1, while dim ChAT cells did not express any of those but do express glycine transporter 1 (Additional file 1: Fig. S1h–k).

### ChAT amacrine cell density decreases in PD

**Fig. 2:**
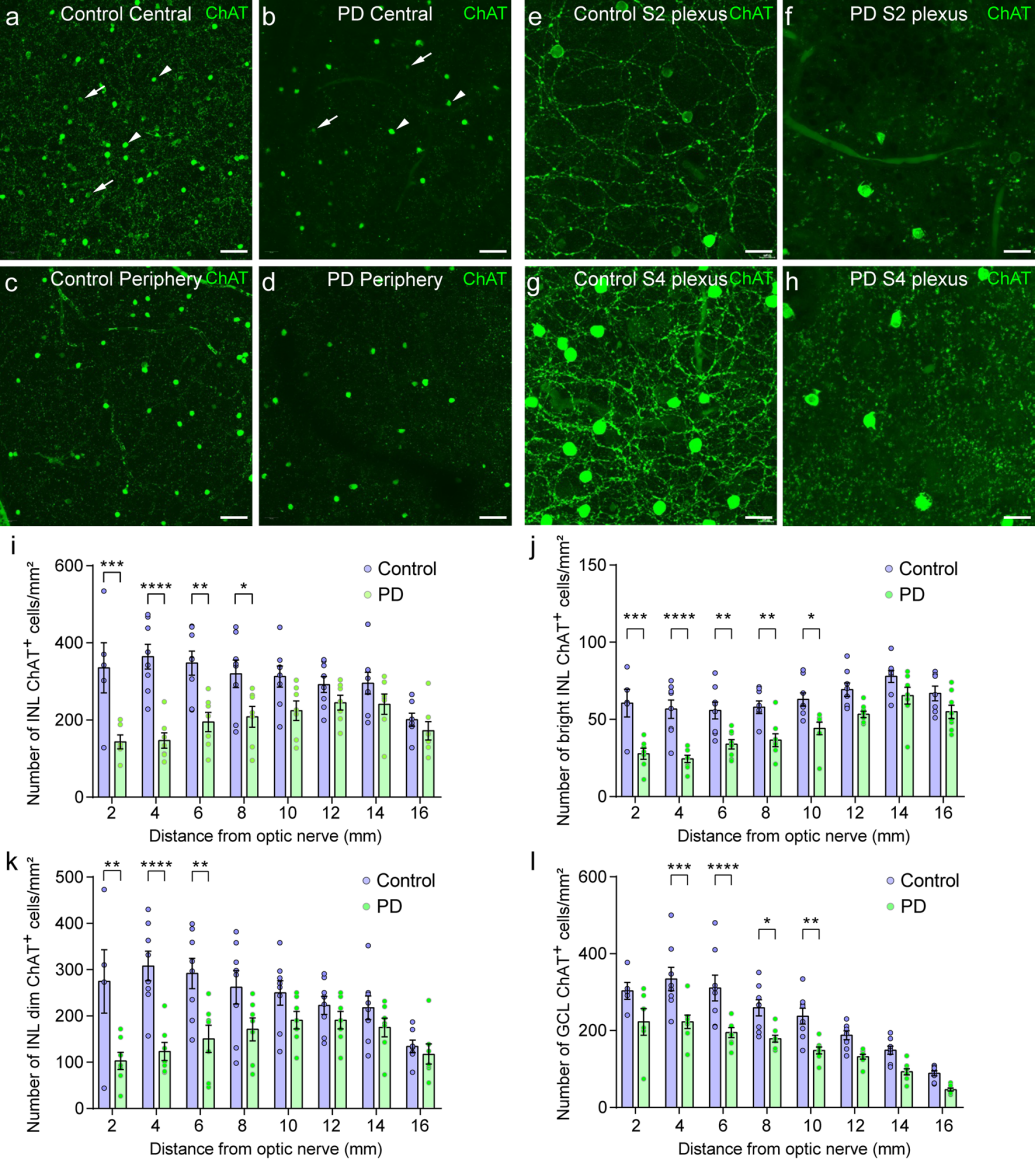
Immunostaining against choline acetyltransferase (ChAT) in wholemount retinas of control and PD patients. **a**–**d** Stack images of representative central and peripheral areas of a retinal wholemount containing both the INL and GCL. **a**, **b** A decrease in the total number of both ChAT-positive cell types: bright (arrowheads) and dim (arrows) could be observed in the central retina in PD compared to control. **c**, **d** The same result could be observed in the peripheral retina. A decrease in the ChAT plexus density in the S2 (**e**, **f**) and in the S4 (**g**, **h**) strata of the IPL could be observed in the PD retinas compared to controls. **i**–**l** Quantification of the total number of ChAT-positive cells in the INL (**i**), the number of bright (**j**) and dim (**k**) ChAT-positive cells in the INL, and the number of ChAT-positive cells in the GCL at 2, 4, 6, 8, 10, 12, 14 and 16 mm from the optic nerve to the inferonasal quadrant in control (*n* = 8) and PD retinas (*n* = 8). Results are presented as mean value ± SEM. Two-way ANOVA with Sidak’s multiple-comparison test. *P < 0.05, **P < 0.01, ***P < 0.001, ****P < 0.0001. *ON* optic nerve. Scale bars: 40 μm (**a**–**d**). 20 μm (**e**–**g**)

Consistent with our previous results, bright (Fig. 2a, b, arrowheads) and dim (Fig. 2a, b, arrows) ChAT cell populations can be observed in retinal wholemounts. All ChAT amacrine cells are distributed at varying densities from the center to the periphery of the retina, being, globally, more abundant in the center. In PD, we found that the total ChAT amacrine cell density and their plexuses were reduced in both central and peripheral regions, which could be observed in stack images of retinal wholemounts containing both the INL and GCL (Fig. 2a–d). Higher-magnification images of both the INL + S2 plexus and the GCL + S4 plexus (Fig. 2e–h) show a reduction in the number of ChAT cells and their plexus density in PD.

To confirm these cell density differences, we quantified the number of ChAT-positive cells in the retinas of control and PD patients. To better compare the two conditions and consider the differences inherent to the retinal region or layer, we quantified separately the number of ChAT amacrine cells in the GCL and in the INL and compared the number of cells at different specific retinal eccentricities. Since we were able to identify two distinct ChAT amacrine cell populations in the human retina, we sought to evaluate both individually. The total number of ChAT amacrine cells in the INL was strongly reduced in PD (*P* < 0.0001) with a pronounced decrease in the central retina, in the areas comprising 2–8 mm from the optic nerve (*P* < 0.05 in each region) to the inferonasal region (Fig. 2i, Additional file 1: Table S2). This was evidenced by the two-way ANOVA interaction between “distance from optic nerve” and “Control/PD” factors (*P* < 0.01). Similarly, bright ChAT cells in the INL were significantly reduced in PD in the areas comprising 2–10 mm from the optic nerve (*P* < 0.05 in each region) (Fig. 2j, Additional file 1: Table S2). The maximum cell loss in the bright ChAT amacrine cells was found in the center, with a decrease of 58% of cells at 4 mm from the optic nerve (Additional file 1: Table S2). The dim ChAT population was also more markedly reduced in the center, showing statistically significant differences in the areas comprising 2–6 mm from the optic nerve (*P* < 0.05 in each region), with a loss of 63% of cells at 2 mm (Fig. 2k, Additional file 1: Table S2). In the GCL, the number of ChAT amacrine cells was also strongly reduced (*P* < 0.0001) in PD, affecting most of the eccentricities analyzed, with a statistically significant reduction from 4 to 10 mm from the optic nerve (*P* < 0.05 in each region), although the tendency was observed throughout the whole retina (Fig. 2l, Additional file 1: Table S2). The decrease of ChAT amacrine cell density in the GCL seemed more uniform throughout the retina, showing a loss of ~ 30–40% of cells in all the regions.

### ChAT amacrine cells receive synaptic contacts from DA cells

**Fig. 3:**
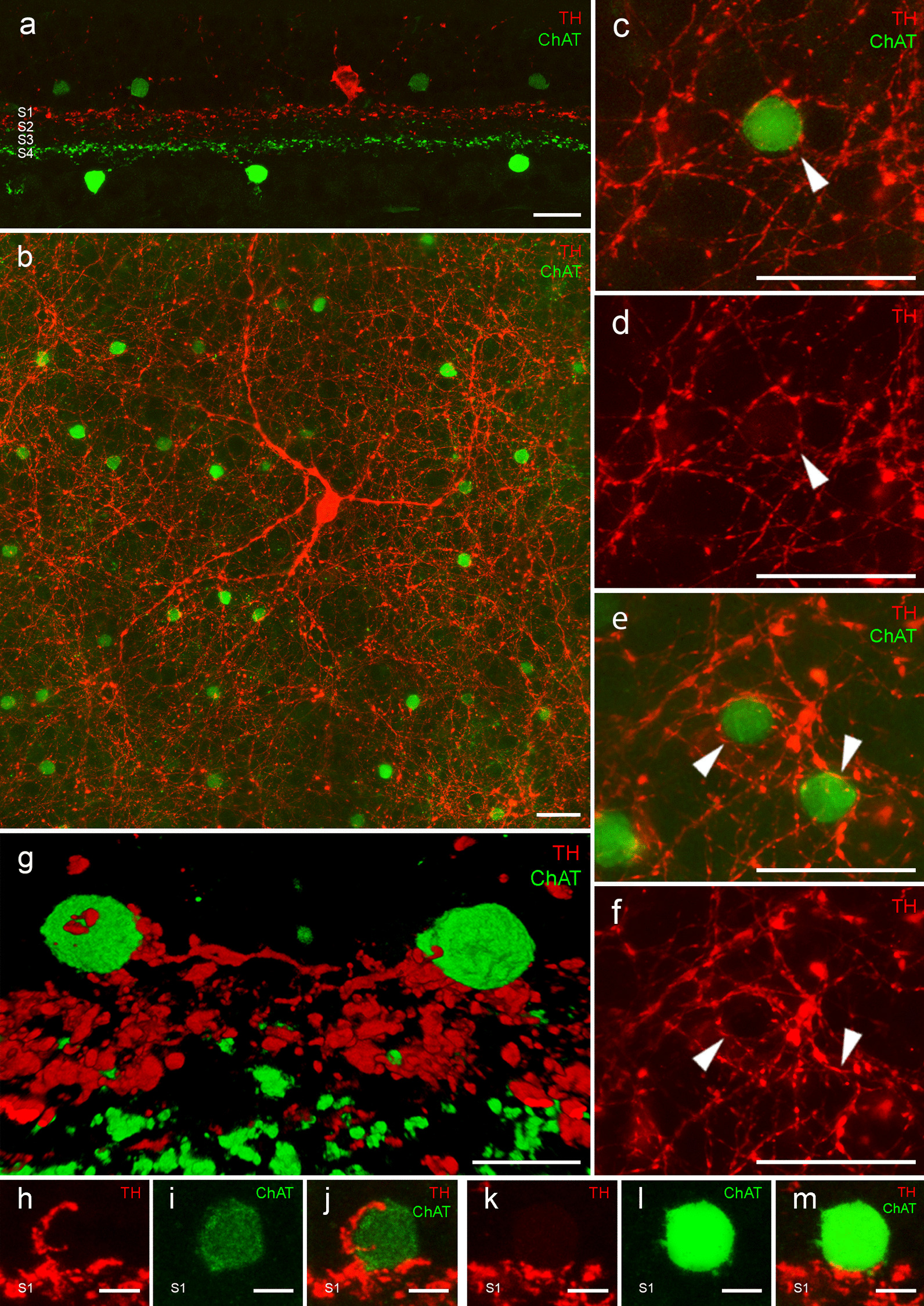
**a** Immunostaining against tyrosine hydroxylase (TH, red) and choline acetyltransferase (ChAT, green) in a section of the human retina. **b** Wholemount human retina with a dopaminergic cell (TH, red) forming the characteristic ring-like structures surrounding ChAT-positive cells (green). **c**–**f** Examples of dopaminergic rings with ChAT-positive cells inside. **g** Three-dimensional view of the connections between the dopaminergic plexus and ChAT-positive cell dendrites and somata. **h–m** Examples of contacts between dopaminergic processes and ChAT-positive cell somata. S1: stratum 1 in the IPL. Scale bars: 20 μm (**a**), 25 μm (**b–f**), 5 μm (**g**), 2.5 μm (**h**–**m**)

As dopaminergic cell degeneration is one of the main characteristics of PD and has also been described in the retina [15], we wondered if the observed ChAT cell loss could be related to the DA cell degeneration in these subjects. For that, we sought to determine if ChAT cells receive synaptic inputs from DA cells. Figure 3a shows the location of dopaminergic and ChAT amacrine cells and their plexus stratification in a retinal section. In retinal wholemounts (Fig. 3b–f), we were able to identify the dopaminergic plexus (Fig. 3b, red) with some of the ChAT cells (green) inside dopaminergic rings. Figure 3c–e shows magnified images of dopaminergic dendrites making contacts (arrowheads) around the ChAT amacrine cells within the ring-like structure. This close contact between dopaminergic and ChAT cells could also be seen in three-dimensional reconstructions of retinal sections (Fig. 3g, Additional file 2: Supplementary video). Other examples of these dopaminergic contacts into ChAT cells in both dim and bright ChAT cells in the INL are shown in Fig. 3h–m, respectively.

DA contacts into ChAT cells do not only occur at the soma level, but also at their dendrites within the IPL. While it may seem that their plexuses are independent (Fig. 3a), we observed some ChAT processes located at the S1 stratum and some dopaminergic processes at the S2 stratum, making it possible for them to connect. A similar thing occurs at the S4 stratum, where dopaminergic neurons can occasionally contact ChAT dendrites in their way towards their ramification at S4/S5 strata.

**Fig. 4:**
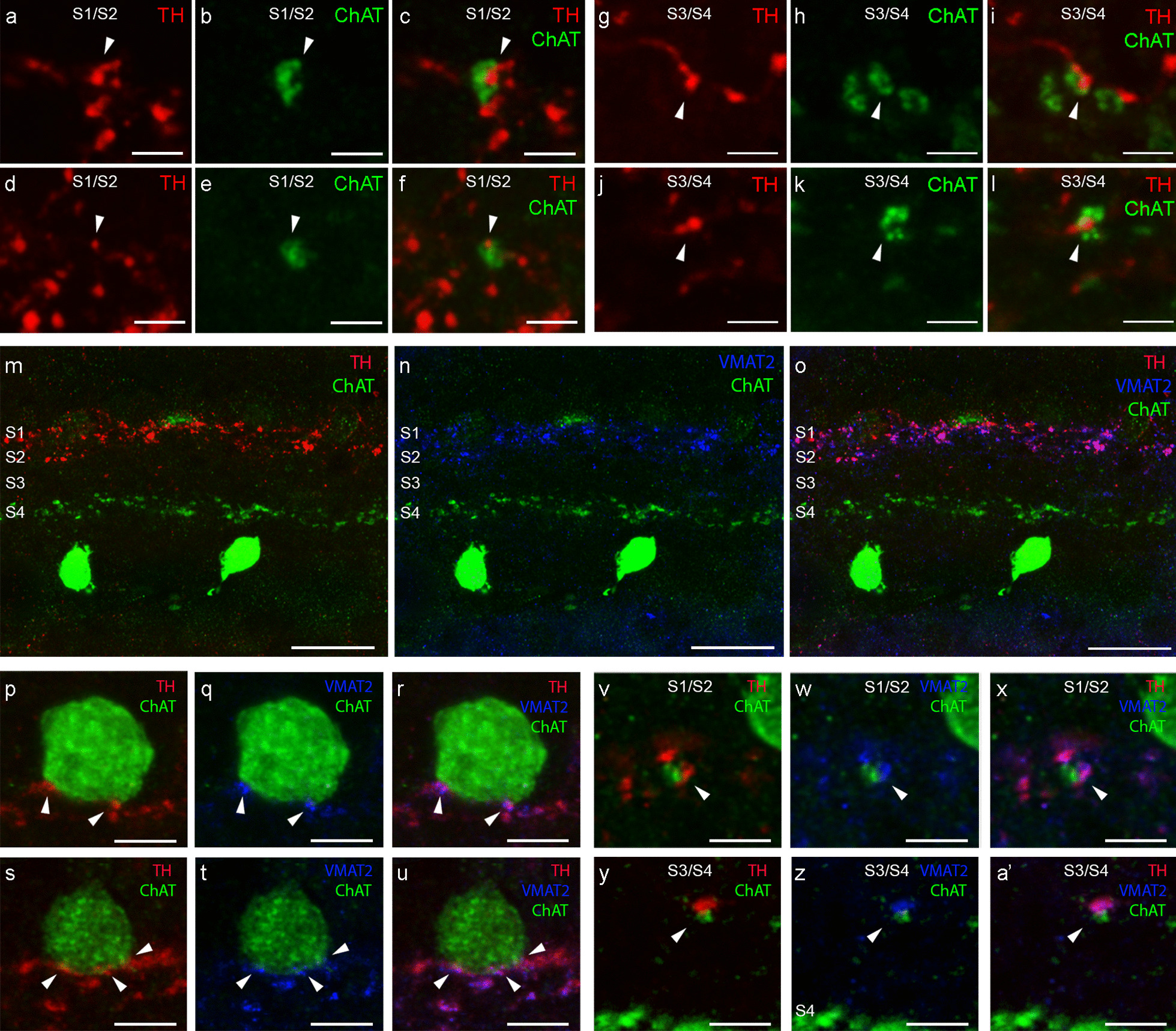
**a**–**l** Immunostaining against tyrosine hydroxylase (TH, red) and choline acetyltransferase (ChAT, green) in a section of the human retina showing examples of connections between ChAT-positive cells and TH-positive cells at the level of S1/S2 (**a**–**f**, arrowheads) and at the level of S3/S4 (**g**–**l**, arrowheads) in the IPL. **m**–**o** Retinal sections immunostained against TH (red), ChAT (green) and vesicular monoamine transporter 2 (VMAT2, blue). **p**–**a**’ Examples of connections between ChAT-positive cell somata (**p**–**u**, arrowheads) and dendrites (**v**–**a**’, arrowheads) with dopaminergic cell dendrites (TH, red) containing VMAT2 (blue). S1/S2: stratum 1 and 2 in the IPL. S3/S4: stratum 3 and 4 in the IPL. Scale bars: 2.5 μm (**a**–**l**). 20 μm (**m**–**o**). 5 μm (**p**–**a**’)

We were able to find IPL contacts (arrowheads) between DA and ChAT amacrine cell dendrites at the S1/S2 (Fig. 4a–f) and S3/S4 (Fig. 4g–l) strata. To confirm that these close contacts are actually synaptic contacts, we stained the sections with antibodies against TH, ChAT, and VMAT2. VMAT2 packages monoamines like dopamine into the synaptic vesicles of the presynaptic terminal [34, 35]. Its presence within the dopaminergic contacts close to ChAT cells indicates that they are true presynaptic sites.

We were able to observe that, in the IPL, the main dopaminergic plexus at S1–S2 and the OFF-ChAT plexus at S2 were close to each other (Fig. 4m–o). In addition, VMAT2 antibody staining is also present in the plexus corresponding to the DA dendrites. Higher-magnification images of the dopaminergic contacts into ChAT cells showed that they contained VMAT2 in the dopaminergic dendrites (arrowheads), at both the ChAT soma (Fig. 4p–u) and the dendrites levels (Fig. 4v–a’). This result confirms that they are true presynaptic terminals and suggests that ChAT cells receive synaptic input from DA cells. These synaptic contacts occurred with bright (Fig. 4p–r) and dim ChAT cells (Fig. 4s–u) at the INL, and with their dendrites located at the S1/S2 (Fig. 4v–x) and S3/S4 IPL strata (Fig. 4y–a’).

Preliminary results from the snRNA-seq data showed that some ChAT cells in both bright and dim clusters express significant levels of the dopamine receptor D4 (Additional file 1: Fig. 1l), adding evidence for the synaptic contacts between dopaminergic and ChAT amacrine cells, and suggesting the D4 receptor as the mediator of those connections.

### Synaptic contacts of dopaminergic cells into ChAT amacrine cells are reduced in PD

**Fig. 5:**
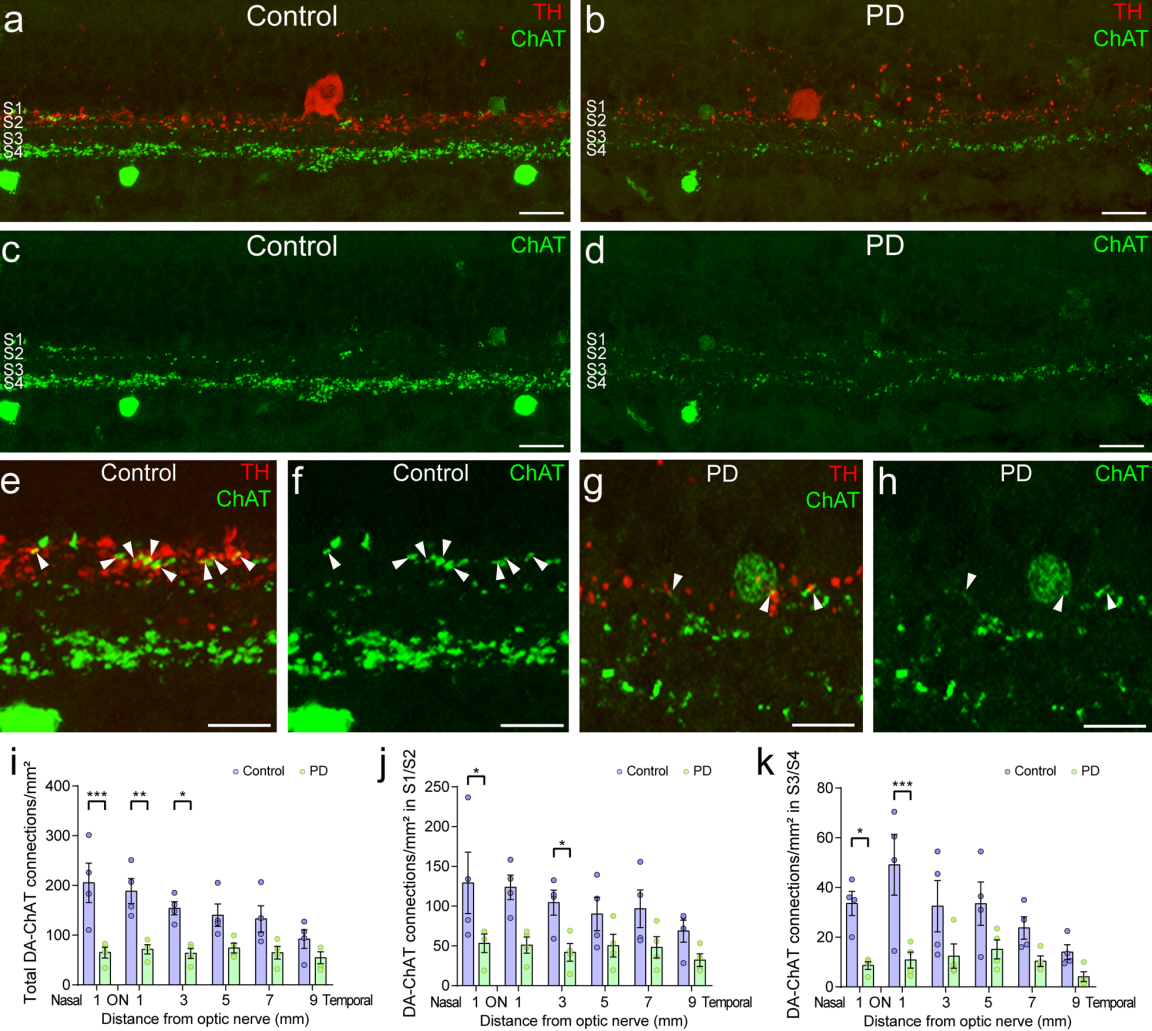
Loss of dopaminergic ChAT contacts in PD. **a**–**h** Immunostaining against tyrosine hydroxylase (TH, red) and choline acetyltransferase (ChAT, green) in retinal sections of control (**a**, **c**) and PD (**b**, **d**) patients, and details of dopaminergic-ChAT connections (arrowheads) in control (**e**, **f**) and PD (**g**, **h**) retinas. **i**–**k** Quantification of the total number of DA-ChAT connections (**i**), the number of DA-ChAT connections in S1/S2 (**j**) and S3/S4 (**k**) strata of the IPL at 1, 3, 5, 7, 9 mm temporal from the optic nerve in control (*n* = 4) and PD retinas (*n* = 5). Two-way ANOVA with Sidak's multiple-comparison test. *P < 0.05, **P < 0.01, ***P < 0.001. ON: optic nerve. Scale bars: 20 μm (**a–d**). 10 μm (**e–h**)

Then, we wondered if the described contacts are affected in PD. A strong reduction in both the DA (red) and ChAT (green) plexus could be observed in PD when compared to control retinas (Fig. 5a–d). Nevertheless, despite the dendritic loss, some of the contacts between DA and ChAT processes were maintained in the remaining dendrites in PD (Fig. 5e–h). The number of those contacts was manually counted in control and PD retinal sections (Fig. 5i–k, Additional file 1: Table S3). The total number of contacts in the S1/S2 and S3/S4 strata was significantly reduced (*P* < 0.05) in PD in the central retina, from the optic nerve to 5 mm temporal to the optic nerve. When each stratum was assessed separately, the differences were still statistically significant at 1 mm nasal and 3 mm temporal from the optic nerve in S1/S2, and at 1 mm from the optic nerve in S3/S4.

**Fig. 6:**
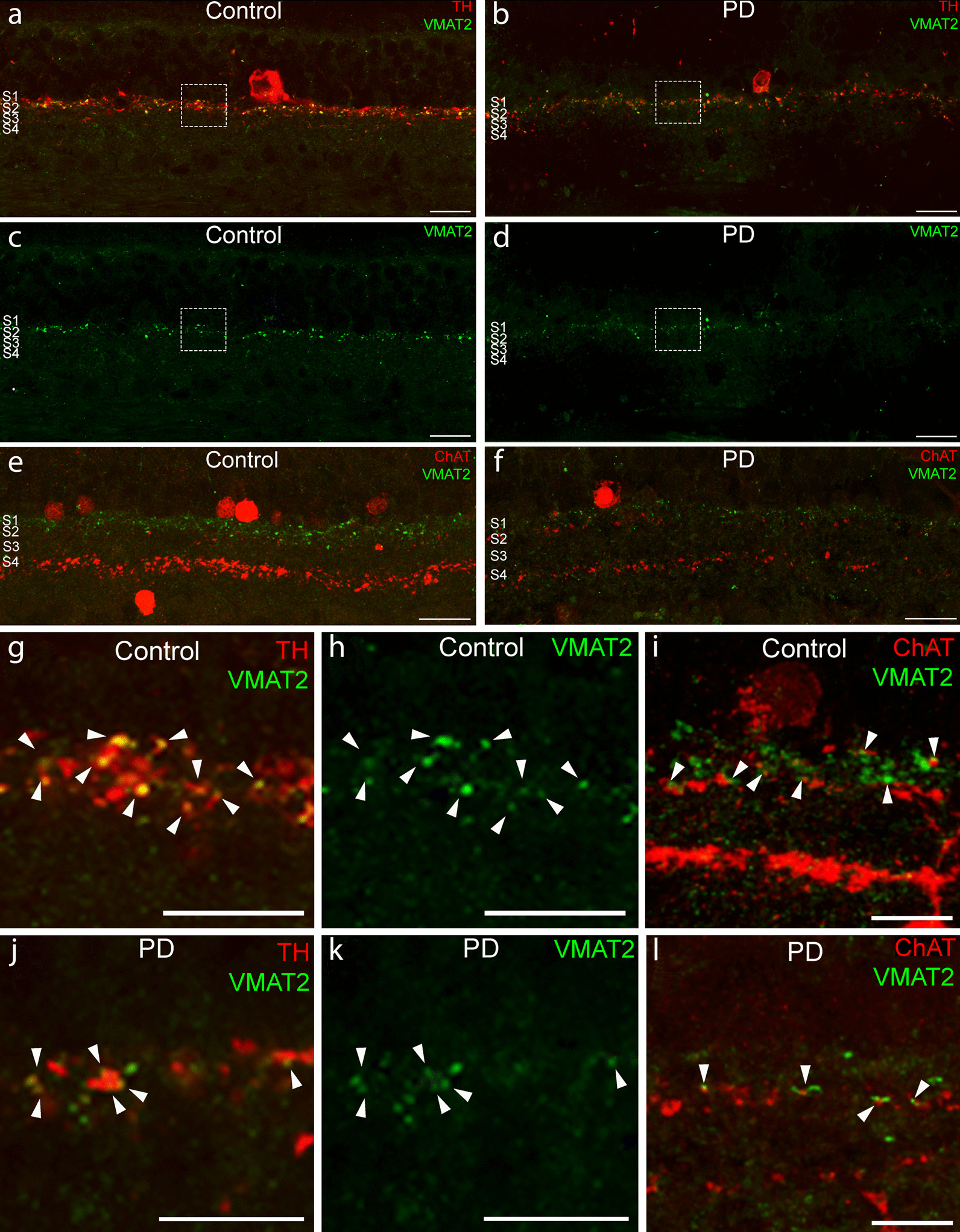
Some synaptic contacts between TH and ChAT cells are preserved in PD. **a–d** Immunostaining against TH (red) and VMAT2 (green) in retinal sections of control (**a**, **c**) and PD (**b**, **d**) patients. Immunostaining against ChAT (red) and VMAT2 (green) in retinal sections of control (**e**) and PD (**f**) patients. Details of TH and VMAT2 colocalization (arrowheads) in control (**g**, **h**) and PD (**j**, **k**) retinas. Insets of ChAT-VMAT2 connections (arrowheads) in control (**i**) and PD (**l**) retinas. Scale bars: 20 μm (**a**–**f**). 10 μm (**g**–**l**)

We also analyzed VMAT2 staining to evaluate if the DA-ChAT contacts contain synaptic vesicles in PD as happens in controls (Fig. 6a–f). Although the number of contacts and the thickness of the plexus containing VMAT2 were decreased in PD, there was VMAT2 within the DA dendrites and in close contact with ChAT processes in the PD retina (Fig. 6g–l). So, despite the observed degeneration, synapses are still happening in PD in the remaining contacts between DA and ChAT cells.

### ChAT density reduction and DA-ChAT synaptic contact loss correlate with dopaminergic degeneration in PD

**Fig. 7:**
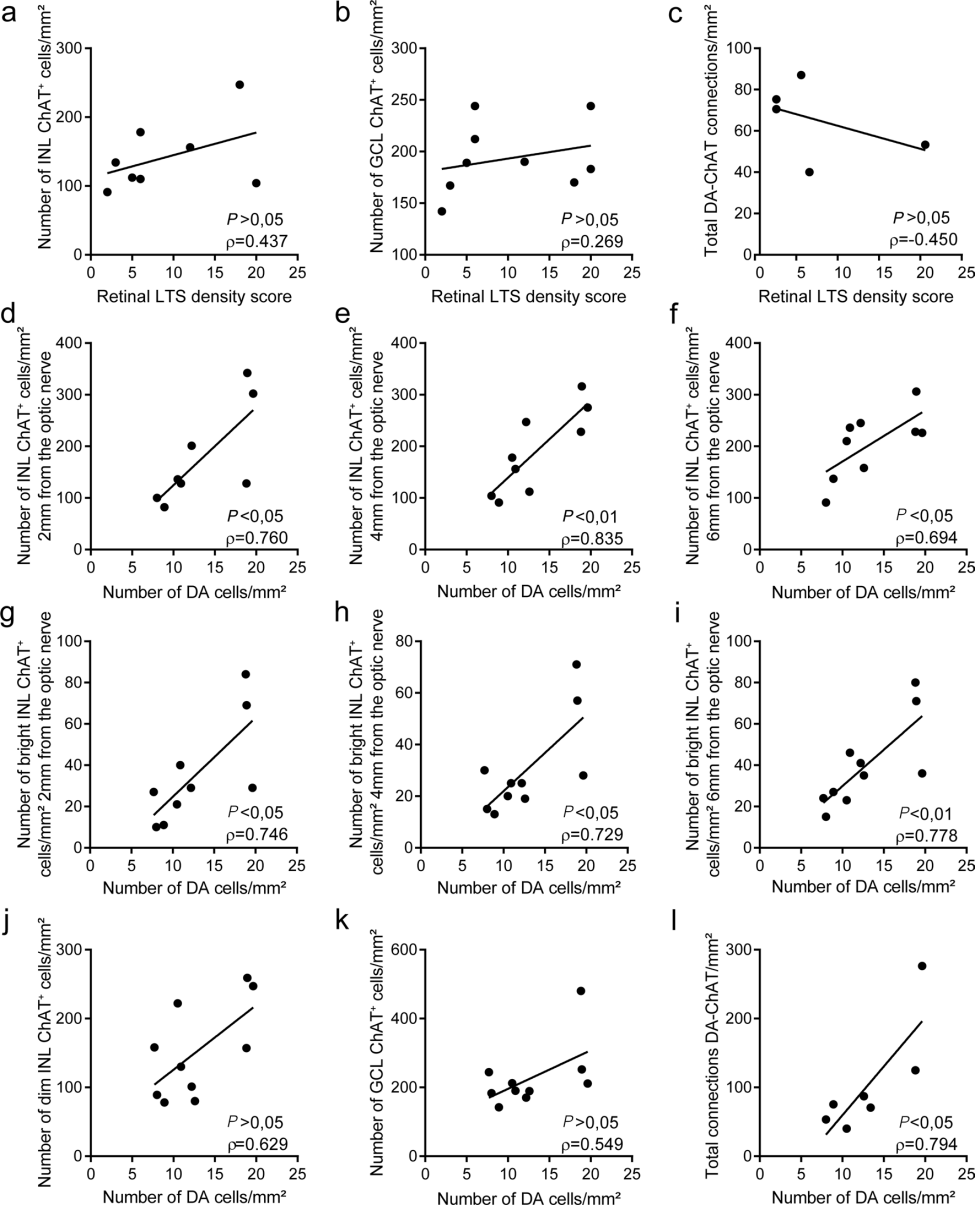
Correlations between the PD degenerative process and our results. **a** Correlation plot between LTS density score in the retina and the number of INL ChAT^+^ cells; Spearman correlation *ρ* = 0.437; *P* > 0.05 (no correlation). **b** Correlation plot between LTS density score in the retina and the number of GCL ChAT^+^ cells; Spearman correlation *ρ* = 0.269; *P* > 0.05 (no correlation). **c** Correlation plot between LTS density score in the retina and the number of DA-ChAT connections in the retina; Spearman correlation *ρ* =  − 0.045; *P* > 0.05 (no correlation). **d** Correlation plot between the number of DA cells in the retina and the number of INL ChAT^+^ cells at 2 mm from the optic nerve; Spearman correlation *ρ* = 0.760; *P* < 0.05. **e** Correlation plot between the number of DA cells in the retina and the number of INL ChAT^+^ cells at 4 mm from the optic nerve; Spearman correlation *ρ* = 0.835; *P* < 0.01. **f** Correlation plot between the number of DA cells in the retina and the number of INL ChAT^+^ cells at 6 mm from the optic nerve; Spearman correlation *ρ* = 0.694; *P* < 0.05. **g** Correlation plot between the number of DA cells in the retina and the number of bright INL ChAT^+^ cells at 2 mm from the optic nerve; Spearman correlation *ρ* = 0.746; *P* < 0.05. **h** Correlation plot between the number of DA cells in the retina and the number of bright INL ChAT^+^ cells at 4 mm from the optic nerve; Spearman correlation *ρ* = 0.729; *P* < 0.05. **i** Correlation plot between the number of DA cells in the retina and the number of bright INL ChAT^+^ cells at 6 mm from the optic nerve; Spearman correlation *ρ* = 0.778; *P* < 0.01. **j** Correlation plot between the number of DA cells in the retina and the number of INL ChAT^+^ cells; Spearman correlation *ρ* = 0.629; *P* > 0.05 (no correlation). **k** Correlation plot between the number of DA cells in the retina and the number of GCL ChAT^+^ cells; Spearman correlation *ρ* = 0.549; *P* > 0.05 (no correlation). **l** Correlation plot between the number of DA cells in the retina and the number of DA-ChAT connections in the retina; Spearman correlation *ρ* = 0.794; *P* < 0.05. Two-tailed Spearman correlation test. LTS: retinal Lewy-type synucleinopathy

The Spearman’s correlation test was performed to further correlate the PD degenerative process and our results. This test revealed no correlation between retinal phosphorylated α-synuclein accumulation (measured as LTS density score) and the degeneration of ChAT amacrine cells (*P* > 0.05, in each comparation) (Fig. 7a–c). Nevertheless, a strong positive correlation between the density of DA and ChAT cells in the INL was observed at 2, 4 and 6 mm from optic nerve (*P* < 0.05, in each region) (Fig. 7d–f). These occurred specifically for the bright (*P* < 0.05, in each region) (Fig. 7g–i) but not the dim population (*P* > 0.05) (Fig. 7j) of INL ChAT. Also, the density of DA cells did not correlate with the GCL ChAT population density (*P* > 0.05) (Fig. 7j). Lastly, we found also a strong correlation between the density of DA and the total connections between DA and ChAT cells (Fig. 7k), indicating that the ChAT degeneration described in this work could be triggered by the loss of synaptic input from DA cells in PD. This was also evidenced by the fact that the percentage of reduction in the number of DA-ChAT connections in PD (54.78% reduction compared to controls) (Additional file 1: Table S4) was higher than the percentage of reduction in the density of ChAT cells (46.11% reduction in INL ChAT cells and 35.15% in GCL cells compared to controls) (Additional file 1: Table S5).

## Discussion

In the present work, we describe a reduction of the density of ChAT amacrine cells in the retinas of PD patients. ChAT amacrine cells are a cell type involved in motion perception, specifically in the process of direction selectivity. In the vertebrate retina, ChAT-expressing amacrine cells have usually been considered starburst amacrine cells, and their somata are located at the INL and at the GCL (displaced) [25–27]. In the control human retina, two types of ChAT amacrine cells with different immunoreactivity intensity and soma location within the INL can be observed. The different expression of parvalbumin, calbindin, GABA and glycine in those cells may indicate that they are two different cell populations. The bright cell type, expressing parvalbumin, calbindin and GABA, and with the typical symmetric mirror location in the INL and GCL, would correspond to the classic ChAT starburst amacrine cells. On the contrary, the dim cell type, not expressing any of those and located only in the INL, not as symmetric pairs, would be a different amacrine cell. We believe that this other type of ChAT cell corresponds to a narrow-field glycinergic amacrine cell described in the ground squirrel retina named Type III and thought to be the A6 amacrine cell [22]. Here we found that the density of both, dim and bright, is reduced in PD compared to controls.

Considering that many studies have reported cholinergic and dopaminergic system alterations in the pathophysiology of PD [36, 37], and that dopamine acts as a wide neuromodulator in the retina, we wondered about the relationship of dopaminergic and ChAT amacrine cells in the human retina. We observed dopaminergic contacts into ChAT cells at their soma (INL) and dendrites, in both dim and bright ChAT populations. Also, in retinal wholemounts at the INL-IPL level, we observed that ChAT cells occupy the characteristic dopaminergic rings where the soma of cells postsynaptic to DA cells are located, a finding that has not been previously described. Although the main postsynaptic cell that is located within those rings is the AII amacrine cell [38, 39], double staining with calretinin (AII cells) and TH (DA cells) reveals that there are rings that do not contain AII cells and thus should contain other amacrine cell type [15]. In addition, besides the AII, other studies in mice have found a population of parvalbulmin-containing amacrine cell inside dopaminergic rings [40]. Both facts support our finding that ChAT cells receive synaptic contacts from DA cells, probably through the D4 receptor as suggested by preliminary snRNA-seq data. We are well aware that the population size of the ChAT cluster was not enough to provide definite conclusions about this issue, but these results agree with the existence of synaptic contacts between DA and ChAT cells that we described using immunohistochemistry. Also, it is reasonable that only a small proportion of the ChAT cells in the clusters express the D4 receptor and our stainings were consistent with it: DA contacts in the soma were only present in a subset of the OFF-ChAT cells. This new finding would imply that dopamine can regulate ChAT cell function in a direct way through their synaptic contacts, similar to its modulation of the AII cells which are also located within dopaminergic rings.

Previous studies in patients and animal models of PD have described the loss of dopaminergic cells and their dendritic plexus in the retina [16, 41–43]. This loss has been related to the loss of synaptic contacts with some of their postsynaptic cells, like the AII amacrine cells or the intrinsically photosensitive melanopsin-containing retinal ganglion cells [15, 16, 42]. Here, we assessed the state of the newly identified dopaminergic input to ChAT amacrine cells in PD and observed that the number of dopaminergic contacts into ChAT cells was reduced. Our results suggest that the retinal dopaminergic system alteration may affect ChAT cells in PD and the observed loss of ChAT amacrine cells in PD might be caused or increased by the dopamine deficiency. If so, experiments using treatments to replace dopamine, which have already been proven to restore visual function in mice [44], could be useful to test if they also protect against the loss of ChAT and other postsynaptic cells. In our study, most of the patients were under dopamine precursor treatments, which could be exerting a partial protective effect against the impairment observed in ChAT cells.

In the same line, there is a positive correlation between the degeneration of DA cells and the DA-ChAT connection decrease, and between the reduction in the density of DA cells and INL ChAT cells. These correlations, together with the fact that the number of connections is more severely reduced than the ChAT density, support the idea that dopaminergic cells make synaptic contacts into ChAT cells. Thus, the degeneration of DA cells reduces the synaptic input to ChAT cells and leads to its degeneration. The correlation occurs specifically with bright but not dim INL ChAT populations, suggesting that starburst amacrine cells (and therefore the motion direction selectivity process) are related to the retinal dopaminergic system. Nevertheless, we could not find the same correlation with GCL ChAT cells, maybe due to the fewer dopaminergic contacts that occur in the ON sublamina of the IPL compared to the OFF sublamina. Lastly, we did not find a correlation between the synucleinopathy in the retina [17, 45] and our results. We believe that the accumulation of phosphorylated α-synuclein which mainly occurs in the retinal ganglion cell layer in humans [17, 45] and the degeneration of the dopaminergic and ChAT amacrine cells might happen as independent processes, triggered by a global retinal dysfunction in PD patients.

One of the main described roles of starburst ChAT amacrine cells is their participation in motion perception. Starburst amacrine cell somas reside in the INL and GCL as two mirror symmetric populations and stratify in the S2 (OFF) and S4 (ON) strata of the IPL, respectively. They make synaptic contacts with direction-selective retinal ganglion cells (DSGCs) [46], and these connections are the most studied origin of direction selectivity in the mouse and rabbit retinas. Through these connections, starburst amacrine cells perform an asymmetric inhibition on these ganglion cells [47]. There are different types of direction-selective ganglion cells depending on the response to the light onset (ON) and offset (OFF) and the different directions of motion. In this sense, we can find four ON–OFF DSGCs [48], which are the most extensively investigated type, three ON DSGCs [48] and one OFF DSGCs [49]. However, while there is a significant amount of evidence on the existence of DSGCs in non-primate mammals [50], their presence in the primate retina had not been confirmed [51] until a recent study that found a single ON–OFF DSGC type [52]. The ON–OFF DSGCs found in non-primates such as mice or rabbits show bistratified dendrites forming plexuses in the ON and OFF sublaminae of the IPL co-stratifying with starburst amacrine dendrites [47]. Interestingly, in the case of the primate retina, ganglion cells with a similar dendritic stratification are also found in the same sublaminae as starburst amacrine cells [53]. This may indicate that there could be a circuitry responsible of motion direction selectivity in the primate retina similar to the non-primate retina, although how the signal is generated and encoded is not fully known. Probably, the lower density of starburst amacrine cells in the INL (bright OFF ChAT cells) compared to the GCL (ON ChAT cells) observed in this work can be attributed to a reduced number of ON–OFF DSGCs to connect to. The reason behind this could be, as we previously mentioned, that only one subtype of ON–OFF DSGC has been described in the primate retina [52] compared to the four subtypes found in the non-primate retina [48]. Furthermore, Kim et al. [52] proposed that a population of monostratified ganglion cells found in the human retina could be the matching subtype for the non-primate ON DSGCs [54] being able to connect with the starburst amacrine cells in the GCL.

Due to their essential participation in this circuit, the loss of ChAT amacrine cells in PD might cause, or partially explain, the described motion perception alterations reported in patients [1, 9, 10]. Also, their degeneration can be partially responsible, together with the loss of other amacrine cells such as DA cells, for the reduced amplitude of electroretinogram oscillatory potentials reported in patients [55]. Thus, their preservation, together with the preservation of the other retinal cell populations affected by PD, would involve a substantial improvement of the patient’s quality of life. In addition, as mentioned before, ChAT displaced amacrine cells residing in the GCL constitute a high percentage of the total cells located at that layer. Structural changes of retinal layers, especially at the inner retina, have been extensively described in PD patients using OCT [14, 56], with the IPL + GCL complex being one of the principal layers affected (57–61). Importantly, most of these studies refer only to a possible loss of retinal ganglion cells to explain those changes, omitting the relevance of displaced amacrine cells in the GCL composition. Considering the high contribution of displaced ChAT cells to the GCL thickness, their loss can also be responsible for the described OCT changes and could be monitored over time using OCT measurements.

In the recent years, increasing evidence supports the affection of the retina in PD pathology. For future directions, it would be useful to evaluate the state of the retina in patients at early stages of the disease to prove its applicability as a PD biomarker. In any case, the ability to study the retina in vivo with non-invasive ophthalmological techniques and the existence of multiple tests to assess retinal structure and visual function make the retina a suitable tissue for helping in PD diagnosis and follow-up. Specifically, considering the loss of ChAT cells in PD that we describe in the present work and its possible effect on motion perception or OCT layer thickness, we propose the use of tasks that assess motion perception or OCT exams to help with PD diagnosis. These parameters, in combination with other visual, sensory, motor, and cognitive tests could help gain a better, more sensitive, and earlier diagnosis and follow-up of the pathology.

## Conclusions

In the present work, we describe a reduction of the density of ChAT amacrine cells in the retina of PD patients, a cell type involved in motion direction selectivity. Also, we report synaptic connectivity between dopaminergic and ChAT amacrine cells in the human retina, and the loss of those contacts in PD. This suggests a relationship between the reduced ChAT density and the previously described degeneration of DA cells in this pathology. In this sense, the loss of starburst amacrine cells could explain or be linked to the impairment on motion perception reported in PD patients.

## Supplementary Information

**Additional file 1: Table S1.** Age and brain pathological stage of the donors at the moment of death. **Table S2.** Quantification of the density of ChAT-positive cells in control (*n* = 8) and PD (*n* = 8) retinas. **Table S3.** Quantification of the density of DA-ChAT connections/mm^2^ in control (*n* = 4) and PD (*n* = 5) retinal sections. **Table S4.** Percentage of reduction in the density of DA-ChAT connections/mm^2^ in PD retinas comparing to control. **Table S5.** Percentage of reduction in the density of ChAT positive cells in PD retinas comparing to control. **Figure S1.** Single-nuclei RNA-sequencing from three adult human retinas.

**Additional file 2: Video 1.** Close contact between dopaminergic and ChAT cells. Three-dimensional reconstruction of a retinal section in which the connections between the dopaminergic plexus and ChAT positive cell dendrites and somata can be observed. View from an immunostaining against tyrosine hydroxylase (TH, red) and choline acetyltransferase (ChAT, green) in a section of the human retina.

## Data Availability

All data generated or analyzed during this study are included in this published article and its supplementary information files.

## Abbreviations

ChAT: Choline acetyltransferase
DA: Dopaminergic amacrine
GCL: Ganglion cell layer
INL: Inner nuclear layer
IPL: Inner plexiform layer
OCT: Optical coherence tomography
PD: Parkinson’s disease
PV: Parvalbumin
TH: Tyrosine hydroxylase
VMAT2: Vesicular monoamine transporter 2
