# Correction: Starburst amacrine cells, involved in visual motion perception, lose their synaptic input from dopaminergic amacrine cells and degenerate in Parkinson’s disease patients

**DOI:** 10.1186/s40035-023-00360-2

**Published:** 2023-05-10

**Authors:** Xavier Sánchez‑Sáez, Isabel Ortuño‑Lizarán, Carla Sánchez‑Castillo, Pedro Lax, Nicolás Cuenca

**Affiliations:** 1 Department of Physiology, Genetics and Microbiology, University of Alicante, San Vicente del Raspeig, Spain; 2 Alicante Institute for Health and Biomedical Research (ISABIAL), Alicante, Spain; 3 Ramón Margalef Institute, University of Alicante, San Vicente del Raspeig, Spain

**Correction: Translational Neurodegeneration (2023) 12:17 (2023)** 10.1186/s40035-023-00348-y

Following publication of the original article [1], the authors reported an error in the article title.

“Lose” was mistakenly typed as “loose” in the original title.

The correct title should read: Starburst amacrine cells, involved in visual motion perception, lose their synaptic input from dopaminergic amacrine cells and degenerate in Parkinson’s disease patients.

The original article [1] has been updated.
