# Supplementary material for: Starburst amacrine cells, involved in visual motion perception, lose their synaptic input from dopaminergic amacrine cells and degenerate in Parkinson’s disease patients
Source: Transl Neurodegener. 2023 Apr 3;12:17. doi: 10.1186/s40035-023-00348-y (PMC10071607; doi:10.1186/s40035-023-00348-y)

**Starburst amacrine cells, involved in visual motion perception, loose their synaptic input from dopaminergic amacrine cells and degenerate in Parkinson’s disease patients.**

Xavier Sánchez-Sáez^#1^, Isabel Ortuño-Lizarán^#1^, Carla Sánchez-Castillo^1^, Pedro Lax^1^ and Nicolás Cuenca^1,2,*^

^#^ Contributed equally

^1^ Department of Physiology, Genetics and Microbiology, University of Alicante, San Vicente del Raspeig, Spain

^2^ Ramón Margalef Institute, University of Alicante, San Vicente del Raspeig, Spain

^*^Corresponding author: cuenca@ua.es

**SUPPLEMENTARY INFORMATION**

**Table S1.** Age and brain pathological stage of the donors at the moment of death.

| **Subject** | **Neuropathological diagnosis** | **Age (years)** | **Unified LB Brain Stage** |  |
| --- | --- | --- | --- | --- |
| 1C | Control | 70 | 0. No Lewy Bodies |  |
| 2C | Control | 72 | 0. No Lewy Bodies |  |
| 3C | Control | 72 | 0. No Lewy Bodies |  |
| 4C | Control | 74 | 0. No Lewy Bodies |  |
| 5C | Control | 79 | 0. No Lewy Bodies |  |
| 6C | Control | 77 | 0. No Lewy Bodies |  |
| 7C | Control | 70 | 0. No Lewy Bodies |  |
| 8C | Control | 75 | 0. No Lewy Bodies |  |
| 9C | Control | 64 | 0. No Lewy Bodies |  |
| 10C | Control | 57 | 0. No Lewy Bodies |  |
| 1PD | PD | 75 | lV. Neocortical |  |
| 2PD | PD | 73 | lll. Brainstem/Limbic |  |
| 3PD | PD | 72 | lV. Neocortical |  |
| 4PD | PD | 70 | lll. Brainstem/Limbic |  |
| 5PD | PD | 79 | lV. Neocortical |  |
| 6PD | PD | 79 | lV. Neocortical |  |
| 7PD | PD | 82 | lV. Neocortical |  |
| 8PD | PD | 69 | lll. Brainstem/Limbic |  |
| 9PD | PD | 88 | lV. Neocortical |  |
| C: Control; PD: Parkinson Disease; LB: Lewy Bodies | | | |  |
|  |  |  |  |  |

**Table S2.** Quantification of the density of ChAT positive cells in control (n=8) and PD (n=8) retinas. 1 mm^2^ was assessed at each eccentricity. Results are presented as mean value ± SEM. ON: optic nerve.

| Distance from ON | ChAT^+^ cells in INL/mm^2^ | | Bright  ChAT^+^ cells in INL/mm^2^ | | Dim  ChAT^+^ cells in INL/mm^2^ | | ChAT^+^ cells in GCL/mm^2^ | |
| --- | --- | --- | --- | --- | --- | --- | --- | --- |
|  | Control | PD | Control | PD | Control | PD | Control | PD |
| 2 mm | 330 ± 60 | 140 ± 20 | 61 ± 9 | 28 ± 4 | 270 ± 70 | 100 ± 20 | 300 ± 20 | 220 ± 30 |
| 4 mm | 360 ± 30 | 150 ± 20 | 57 ± 6 | 24 ± 2 | 310 ± 30 | 120 ± 20 | 330 ± 30 | 220 ± 20 |
| 6 mm | 350 ± 30 | 180 ± 20 | 56 ± 5 | 33 ± 3 | 290 ± 30 | 150 ± 30 | 310 ± 30 | 190 ± 10 |
| 8 mm | 320 ± 30 | 190 ± 20 | 58 ± 4 | 36 ± 4 | 260 ± 40 | 170 ± 20 | 260 ± 20 | 179 ± 9 |
| 10 mm | 310 ± 30 | 210 ± 30 | 63 ± 4 | 44 ± 4 | 250 ± 30 | 190 ± 20 | 240 ± 20 | 148 ± 9 |
| 12 mm | 290 ± 20 | 220 ± 20 | 69 ± 4 | 53 ± 2 | 220 ± 20 | 190 ± 20 | 190 ± 10 | 132 ± 6 |
| 14 mm | 290 ± 30 | 240 ± 20 | 78 ± 4 | 65 ± 5 | 220 ± 20 | 170 ± 20 | 150 ± 10 | 93 ± 8 |
| 16 mm | 200 ± 20 | 170 ± 20 | 67 ± 5 | 55 ± 4 | 130 ± 10 | 120 ± 20 | 89 ± 6 | 46 ± 4 |

**Table S3.** Quantification of the density of DA-ChAT connections/mm^2^ in control (n=4) and PD (n=5) retinal sections. 1 mm^2^ was assessed at each eccentricity. Results are presented as mean value ± SEM. ON: optic nerve.

| Region | Distance from ON | Total DA-ChAT connections/mm^2^ | | DA-ChAT connections/mm^2^ in S1/S2 | | DA-ChAT connections/mm^2^ in S3/S4 | |
| --- | --- | --- | --- | --- | --- | --- | --- |
|  |  | Control | PD | Control | PD | Control | PD |
| Nasal | 1 mm | 200 ± 40 | 60 ± 10 | 130 ± 40 | 50 ± 10 | 33 ± 5 | 8 ± 1 |
| ON | |  | |  | |  | |
|  | 1 mm | 190 ± 20 | 71 ± 9 | 120 ± 10 | 50 ± 10 | 50 ± 10 | 10 ± 3 |
|  | 3 mm | 150 ± 10 | 60 ± 10 | 100 ± 10 | 40 ± 10 | 30 ± 10 | 12 ± 5 |
|  | 5 mm | 140 ± 20 | 70 ± 10 | 90 ± 20 | 50 ± 10 | 33 ± 9 | 15 ± 4 |
|  | 7 mm | 130 ± 30 | 60 ± 10 | 100 ± 20 | 50 ± 10 | 24 ± 4 | 10 ± 2 |
| Temporal | 9 mm | 90 ± 20 | 50 ± 10 | 70 ± 10 | 32 ± 8 | 14 ± 3 | 4 ± 2 |

**Table S4.** Percentage of reduction in the density of DA-ChAT connections/mm^2^ in PD retinas comparing to control. ON: optic nerve.

|  | Distance from ON | Reduction of total DA-ChAT connections/mm^2^ in PD (%) | Reduction of DA-ChAT connections/mm^2^ in S1/S2 in PD (%) | Reduction of DA-ChAT connections/mm^2^ in S3/S4 in PD (%) |
| --- | --- | --- | --- | --- |
| Nasal | 1 mm | 69.00 | 61.54 | 72.73 |
| ON | |  |  |  |
|  | 1 mm | 64.21 | 59.17 | 80.00 |
|  | 3 mm | 60.00 | 60.00 | 66.67 |
|  | 5 mm | 49.29 | 44.44 | 60.61 |
|  | 7 mm | 46.15 | 50.00 | 58.33 |
| Temporal | 9 mm | 40.00 | 52.86 | 71.43 |
| Mean | | 54.78 | 54.67 | 68.29 |

**Table S5.** Percentage of reduction in the density of ChAT positive cells in PD retinas comparing to control. ON: optic nerve.

| Distance from ON | Reduction of INL ChAT^+^ cells in PD (%) | Reduction of bright INL ChAT^+^ cells in PD (%) | Reduction of dim INL ChAT^+^ cells in PD (%) | Reduction of GCL ChAT^+^ cells in PD (%) |
| --- | --- | --- | --- | --- |
| 2 mm | 57.58 | \| 59.02 \| \| --- \| | \| 62.96 \| \| --- \| | 30.00 |
| 4 mm | 61.11 | \| 59.65 \| \| --- \| | \| 61.29 \| \| --- \| | 36.36 |
| 6 mm | 48.57 | \| 42.86 \| \| --- \| | \| 51.72 \| \| --- \| | 38.71 |
| 8 mm | 37.50 | \| 37.93 \| \| --- \| | \| 34.62 \| \| --- \| | 31.92 |
| 10 mm | 25.81 | \| 26.98 \| \| --- \| | 24.00 | 38.75 |
| Mean | 46.11 | 45.29 | 46.92 | 35.15 |

**Figure S1.** Single-nuclei RNA-sequencing from three adult human retinas. **a** UMAP representation of the different clusters with all cells showing in a red square our cluster of interest. **b**-**d** The cells within the ChAT cluster (red square) express CHAT (**b**) and common amacrine markers as PAX6 (**c**) or ELAVL3 (**d**). **e** DimPlot of the re-clustered ChAT population showing two independent clusters corresponding to the bright and dim ChAT populations. **f** Dimplot and (**g**) violin plot show the expression of CHAT in all the cells. **h**-**k** Only bright ChAT cells express CALB1 (calbindin) (**h**), SLC6A1 (GABA transporter 1) (**i**), and PVALB (parvalbumin) (**j**), while dim cells don’t express any of those but express SLC6A9 (Glycine transporter 1) (**k**). **l** Some cells in both the dim and bright clusters express the dopamine receptor 4 (DRD4).

**Supplementary Video 1.** Close contact between dopaminergic and ChAT cells. Three-dimensional reconstruction of a retinal section in which the connections between the dopaminergic plexus and ChAT positive cell dendrites and somata can be observed. View from an immunostaining against tyrosine hydroxylase (TH, red) and choline acetyltransferase (ChAT, green) in a section of the human retina.


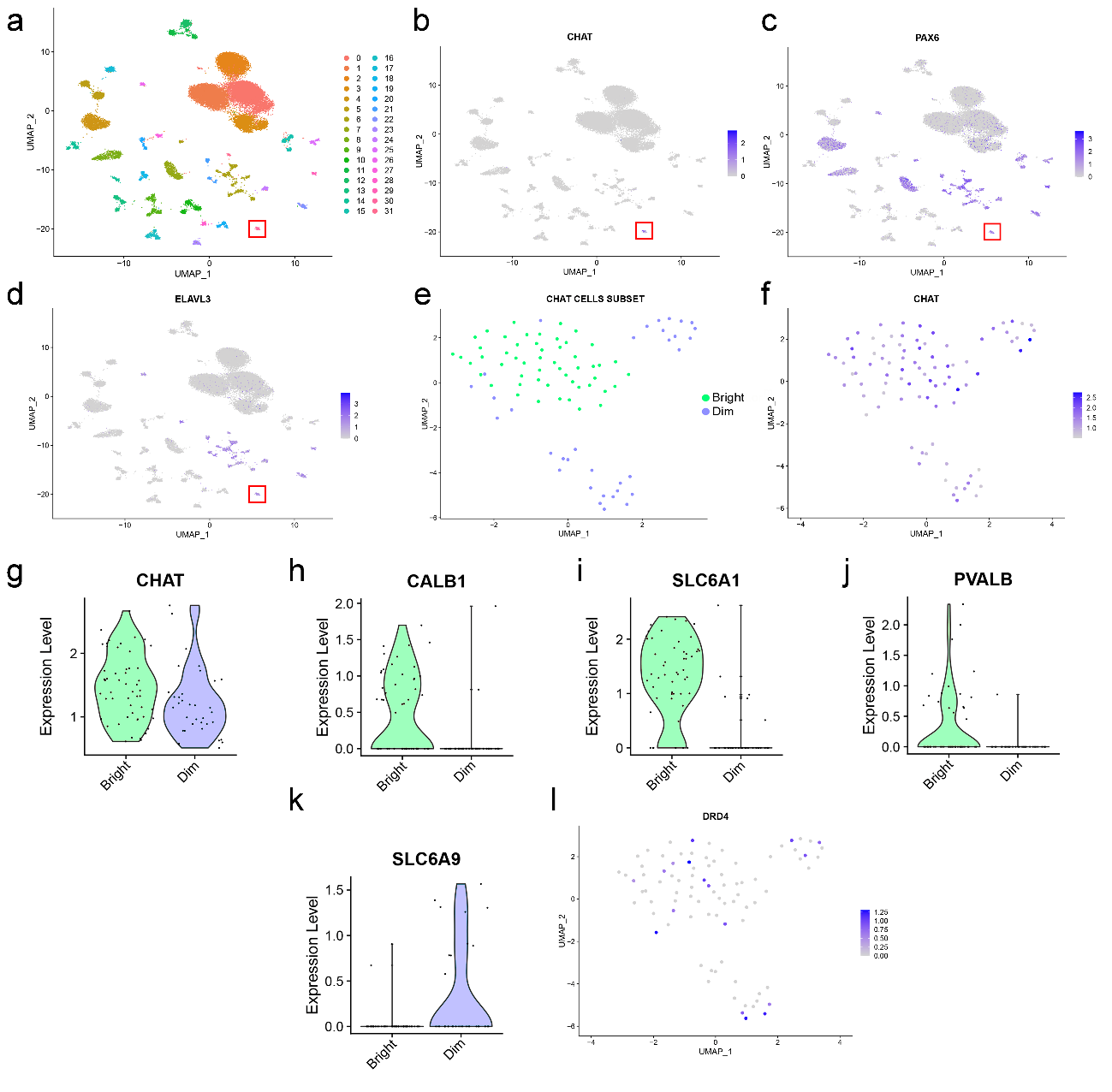

Supplement: Supplementary file 1 — Additional file 1: Table S1. Age and brain pathological stage of the donors at the moment of death. Table S2. Quantification of the density of ChAT-positive cells in control (n = 8) and PD (n = 8) retinas. Table S3. Quantification of the density of DA-ChAT connections/mm2 in control (n = 4) and PD (n = 5) retinal sections. Table S4. Percentage of reduction in the density of DA-ChAT connections/mm2 in PD retinas comparing to control. Table S5. Percentage of reduction in the density of ChAT positive cells in PD retinas comparing to control. Figure S1. Single-nuclei RNA-sequencing from three adult human retinas. [file 40035_2023_348_MOESM1_ESM.docx]
